# Supplementary material for: Peptidases Are Potential Targets of Copper(II)-1,10-Phenanthroline-5,6-dione Complex, a Promising and Potent New Drug against Trichomonas vaginalis
Source: Pathogens. 2023 May 22;12(5):745. doi: 10.3390/pathogens12050745 (PMC10224193; doi:10.3390/pathogens12050745)
Supplement: Supplementary file 1 [file pathogens-12-00745-s001.zip › pathogens-2332264-supplementary-figures.pdf]

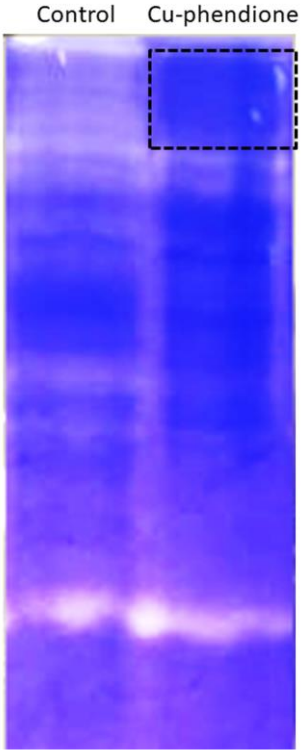

Figure S1. Light microscopy of cytolysis assay with HMVII lineage after copper-phendione-treated *Trichomonas vaginalis* isolates ATCC 30236, TV-LACH4, TV-LACM15, TV-LACM22. Cytolysis assay was carried out as described in Material and Methods section. HMVII means cells only; HMVII + Triton X-100: positive control and total disruption of cell monolayer, as expected. In sequence, at the panel left side: each *T. vaginalis* isolate in contact with HMVII cells disrupted the monolayer; at the panel right side: cytolysis was inhibited by Cu-phendione treatment. Asterisk indicates HMVII cells; arrow indicates *T. vaginalis* trophozoite. Pictures were taken from representative microscopy fields under 400× magnification.

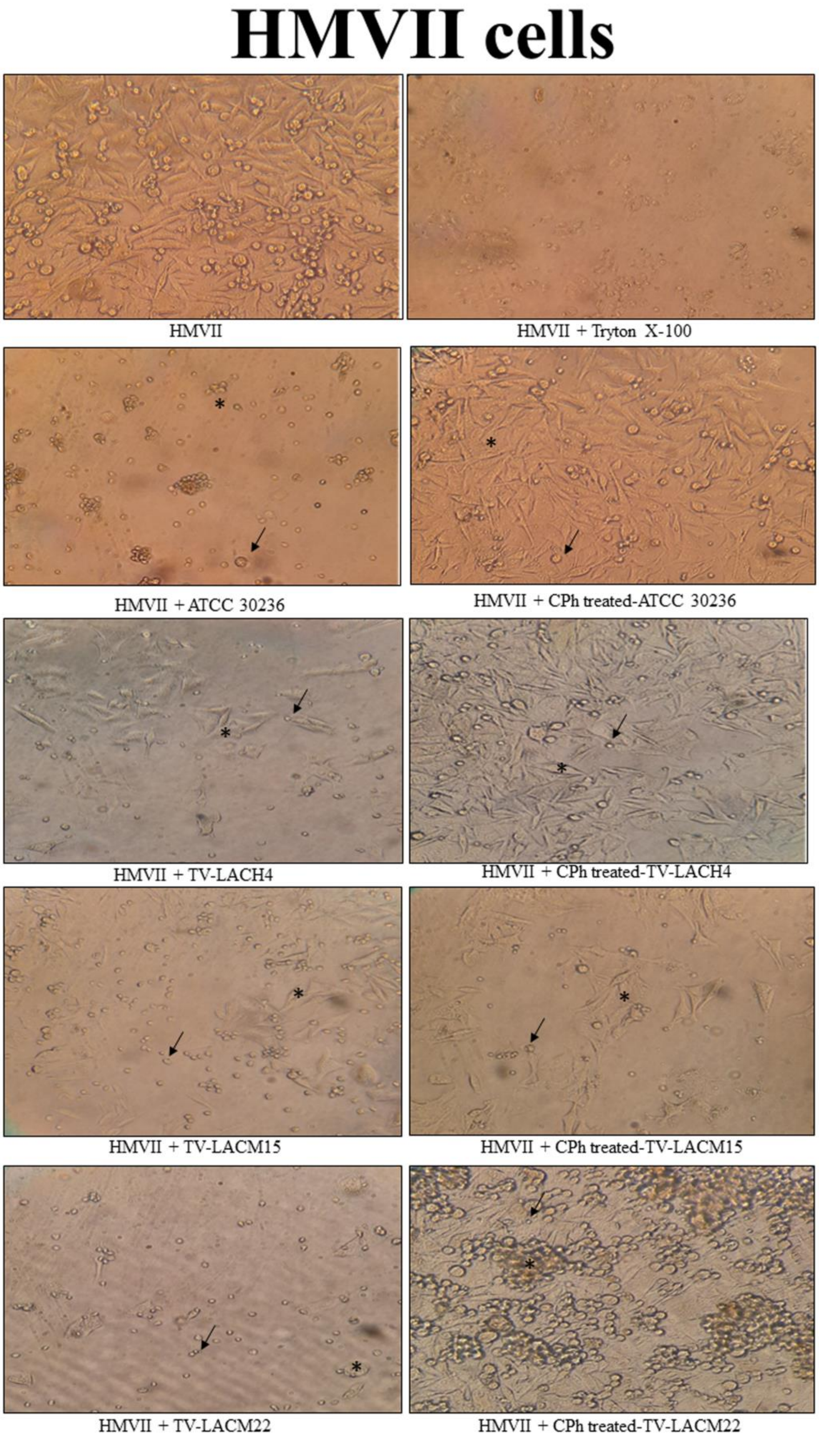

Figure S2. Light microscopy of cytolysis assay with VERO lineage after copper-phendione-treated *Trichomonas vaginalis* isolates ATCC 30236, TV-LACH4, TV-LACM15, TV-LACM22. Cytolysis assay was carried out as described in Material and Methods section. VERO means cells only; VERO + Triton X-100:

positive control and total disruption of cell monolayer, as expected. In sequence, at the panel left side: each *T. vaginalis* isolate in contact with VERO cells disrupted the monolayer; at the panel right side: cytolysis was inhibited by Cu-phendione treatment. Asterisk indicates VERO cells; arrow indicates *T. vaginalis* trophozoite. Pictures were taken from representative microscopy fields under 400× magnification.
